# Demographic and environmental factors associated with the distribution of *Aedes albopictus* in Cameroon

**DOI:** 10.1111/mve.12619

**Published:** 2022-10-20

**Authors:** Tiago Canelas, Edward Thomsen, Basile Kamgang, Louise A. Kelly‐Hope

**Affiliations:** ^1^ Department of Vector Biology Liverpool School of Tropical Medicine Liverpool UK; ^2^ Medical Research Council Epidemiology Unit University of Cambridge Cambridge UK; ^3^ Department of Medical Entomology Centre for Research in Infectious Diseases Yaoundé Cameroon; ^4^ Department of Livestock and One Health Institute of Infection, Veterinary and Ecological Sciences, University of Liverpool Liverpool UK

**Keywords:** *Aedes*, *Aedes albopictus*, Arbovirus, Cameroon

## Abstract

*Aedes*‐transmitted arboviruses have spread globally due to the spread of *Aedes aegypti* and *Aedes albopictus*. Its distribution is associated with human and physical geography. However, these factors have not been quantified in Cameroon. Therefore, the aim was to develop an *Ae*. *albopictus* geo‐referenced database to examine the risk factors associated with the vector distribution in Cameroon. Data on the *Ae*. *albopictus* presence and absence were collated and mapped from studies in published scientific literature between 2000 and 2020. Publicly available earth observation data were used to assess human geography, land use and climate risk factors related to the vector distribution. A logistic binomial regression was conducted to identify the significant risk factors associated with *Ae*. *albopictus* distribution. In total, 111 data points were collated (presence = 87; absence = 24). Different data collection methods and sites hindered the spatiotemporal analysis. An increase of one wet month in a year increased the odds of *Ae*. *albopictus* presence by 5.6 times. One unit of peri‐urban area increased the odds by 1.3 times. Using publicly available demographic and environmental data to better understand the key determinants of mosquito distributions may facilitate appropriately targeted public health messages and vector control strategies.

## Graphical abstract

There has been an introduction of *Aedes albopictus* over the last two decades from the southern region in the first decade to the northern region towards 2019.
Aridness is the main environmental factor keeping *Ae. albopictus* outside the northern region of the country.
The transition from rural to urban areas allows for a better environment for the *Ae. albopictus*.

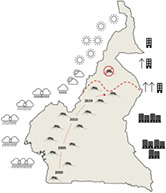

## INTRODUCTION

The *Aedes*‐transmitted arboviruses such as dengue, yellow fever, chikungunya and Zika have rapidly spread globally in the last decade (Kraemer et al., 2015; WHO, 2017, 2021). These arboviruses are of significant public health concern, causing widespread morbidity and mortality, and their expansion to the sub‐Saharan Africa (SSA) region poses a major threat (Weetman et al., 2018). It is estimated that around 70% of the sub‐Saharan African population is at risk of one of these arboviruses; however, the true burden is difficult to determine given the challenges of differential diagnosis in malaria co‐endemic and resource‐poor settings (Weetman et al., 2018).

The expansion of arboviral infections and disease is related to the distributions of the two main mosquito vectors, *Aedes aegypti* (=*Stegomyia aegypti*) and *Aedes albopictus* (=*Stegomyia albopicta*). Native to the African continent, *Ae*. *aegypti* is the main vector of these arboviruses globally (Moore et al., 2013). *Ae*. *albopictus* is native to the South‐East Asian forests and was introduced to the African continent less than 30 years ago through the international commercialization of used tyres, and is now found in many countries across the continent (Bonizzoni et al., 2013; Brady & Hay, 2020; Weetman et al., 2018).

The geographical distribution of these two vectors is driven by human and physical geography, and a wide range of environmental factors (Brady et al., 2014; Cunze et al., 2016; Dickens et al., 2018; Kraemer et al., 2015, 2019). While these two vectors may coexist in the same areas (Brady & Hay, 2020; Paupy et al., 2010; Tedjou et al., 2020), they have been found to have different characteristics on a micro‐level (Egid et al., 2021). For example, both species breed in containers, however *Ae*. *aegypti* prefers human‐made containers in urban areas, while *Ae*. *albopictus* prefers containers surrounded by the presence of vegetation frequently found in peri‐urban and rural areas (Brady et al., 2014; Brady & Hay, 2020; Egid et al., 2021; Kamgang et al., 2010; Kraemer et al., 2015; Tedjou et al., 2020).

*Aedes albopictus* is considered to be a secondary vector for dengue in Africa (Brady & Hay, 2020), but it is the main vector for chikungunya in Central Africa in recent years (Kamgang et al., 2018; Paupy et al., 2010). However, its importance may increase with the rapidly expanding peri−/urbanization taking place across SSA (United Nations, Department of Economic and Social Affairs, 2019). Changes in the urban–rural interface, land use and land cover (LULC), and climate over time and space are important to monitor as they may influence vector distributions and risk (Ali et al., 2017; Burkett‐Cadena & Vittor, 2018; Kalbus et al., 2021; Kamal et al., 2018; Ryan et al., 2018). For example, a lack of rainfall has been found to make larval and pupal stages of *Ae*. *albopictus* vulnerable to desiccation, except in areas where human‐made breeding containers were available providing opportunities for breeding (Waldock et al., 2013).

Defining the human geography, land use patterns and climate envelope where the *Ae*. *albopictus* is present may help to identify key risk factors and target control measures. Recently in Cameroon, two main studies provided an update on the distribution of *Ae*. *aegypti* and *Ae*. *albopictus* with geographical coordinates available for mapping (Simard et al., 2005; Tedjou et al., 2019). The later update in 2017 describes the distribution of *Ae*. *albopictus* being restricted to a certain latitude, that is, under 6° N, while *Ae*. *aegypti* was found to occur across the entire country (Tedjou et al., 2019). However, a study in 2018 identified *Ae*. *albopictus* at a higher latitude near the city of Garoua at 9.3° N for the first time (Rodrigue Simonet et al., 2020), which is in line with global distribution predictions (Kraemer et al., 2015).

The factors influencing the geographical parameters of *Ae*. *albopictus* in Cameroon have not been examined but may be related to a combination of demographic (i.e., population and urbanization) and environmental (land cover, vegetation, temperature and rainfall) characteristics (Dickens et al., 2018; Kraemer et al., 2015), especially as their distribution may be affected in the future by a changing climate (Kamal et al., 2018). A better understanding of these risk factors may help predict new areas vulnerable to establishment of *Ae*. *albopictus* and direct the management of vector control as a means to prevent arboviral diseases. Therefore, the aim of the paper was to extend the work on the recent update (Tedjou et al., 2019) and develop a geo‐referenced vector database based on all publicly available records of *Ae*. *albopictus*, and to identify the potential demographic and environmental risk factors associated with the distribution in Cameroon.

## METHODS

### Vector data sources

First, a geo‐referenced database on *Ae*. *albopictus* in Cameroon was developed. Information from a review up to 2010 was collated (Kraemer et al., 2015) and then a comprehensive literature search was conducted using the online sources of PubMed and Web of Science to identify additional articles with data collected between 2000 and 2020. Search terms, and combinations thereof, included Cameroon, arbovirus, *Aedes*, *Ae. albopictus*, *Ae. aegypti*, dengue and chikungunya (Additional material 1). Further articles were sought from references listed within the identified articles, and then from references within those articles.

For each article the following information was collated into a database; name of collection site (i.e., village, town) as stated in the article; the local administrative area as defined by recent boundaries obtained from United Nations Office for the Coordination of Humanitarian Affairs office (OCHA) in Cameroon; the year of data collection (based on study start month); latitude and longitude of study site; vector presence or absence; and publication information.

### Risk factor data sources

To examine demographic and environmental factors associated with distribution of *Ae*. *albopictus*, data from remote sensed satellite‐derived sources were used. Specifically, data were identified and examined in relation to four broad risk categories: human geography, landscape, vegetation, and climate, and the specific variables and data sources are listed in Table 1.

**TABLE 1:** Summary of demographic and environmental variables

Variable	Spatial resolution	Temporal resolution	Unit of measure	Description	Source
Human geography					
Population	100 m	Yearly	People per pixel	People per pixel adjusted UN estimated	WorldPop
Population density	1 km	Yearly	People per pixel	Number of people per pixel divided by pixel size	WorldPop
Degree of urbanization	1 km	2000 and 2015	Urban, peri‐urban or rural	Classification on urban, peri‐urban or rural area within the pixel	GHSL
Landscape					
Elevation	30 m	‐	Metres	Elevation above sea level	SRTM
Land cover	500 m	Yearly	Land cover types	Land cover types. The authors used the Annual International Geosphere‐Biosphere Programme (IGBP) classification	MODIS
Vegetation					
Enhanced Vegetation Index	250 m	16 days	EVI	Yearly averaged EVI by pixel	MODIS
Forest canopy height	30 m	2019	Metres	Footprint‐based measurements of vegetation structure, including forest canopy height in metres	UMD GLAD
Forest loss	30 m	Yearly	Percentual	Changes of trees (>5 m) in a pixel over a year	UMD GLAD
Climate					
Land surface temperature	1 km	8 days	Degrees Celsius	Monthly and yearly averaged temperature by pixel	MODIS
Rainfall	5 km	Daily	mm^3^	Monthly and yearly total rainfall by pixel	CHIRPS
Wet months	5 km	Monthly	mm^3^	Number of months with more rainfall than 60 mm^3^. Köppen classification	CHIRPS

*Note*: Full variable sources in Additional File 2.

### Mapping and analysis

All geo‐referenced data were imported into the mapping software QGIS 3.16 (https://qgis.org/). First, all collection sites with information on the presence or absence of *Ae*. *albopictus* were mapped into two different decades (2000–2010 and 2011–2020) to examine patterns over time, using the available latitude and longitude coordinates. If the paper did not report the sampling coordinates, the authors generated the centroid coordinates of the lowest level administrative boundary associated with the study areas in the paper. In Cameroon, the lowest level administrative boundaries were sourced from OCHA with a median area of 615 km^2^.

Due to the different spatial resolution and the probable inaccuracy of some data points, the authors created a 3 km buffer around each point using the QGIS geoprocessing spatial tool, and risk factor data were extracted within the buffer using the Zonal Statistics Raster Analysis tool. For each variable, the values were aggregated within the buffer for the analysis.

The remote sensed datasets were processed in Google Earth Engine. All environmental data were exported to statistical software R 4.0.1 (https://www.r-project.org/) for descriptive and statistical analysis. To explore which variables were associated with the presence/absence of *Ae*. *albopictus*, the authors compared them using the Mann–Whitney non‐parametric test (*p* ≤ 0.05 significance and corrected by Bonferroni). Then, a binomial logistic regression model was constructed using variance inflation factor (vif) to determine multicollinearity (excluded vif >5) of variables and stepwise analysis by AIC to select among the 13 variables collected for the final model.

## RESULTS

### *Collation of* Ae*.* albopictus *data*

**FIGURE 1:**
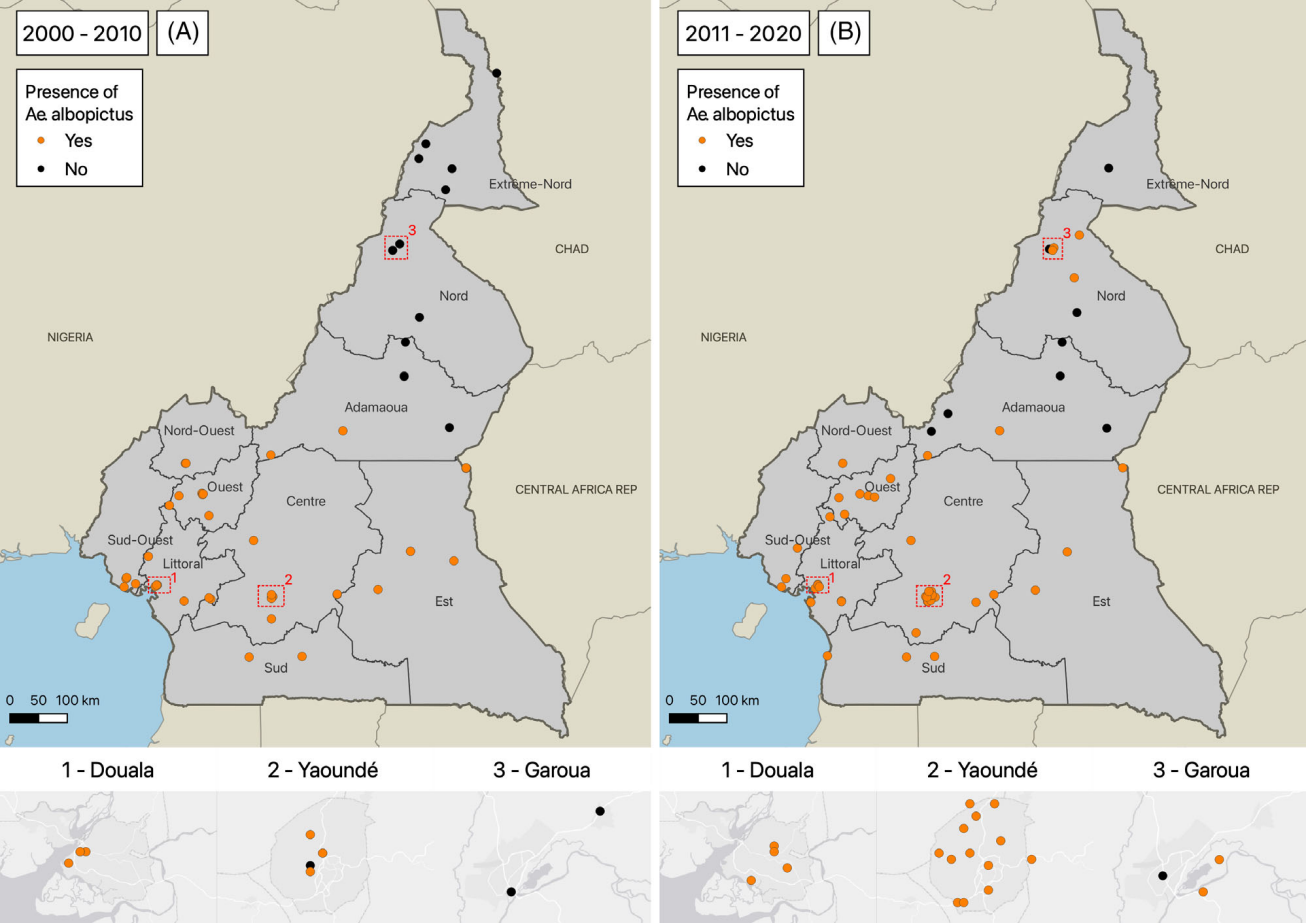
Distribution of *Ae*. *albopictus*. (a) 2000–2010. (b) 2011–2020

In total, 12 articles reporting information on the presence or absence of *Ae*. *albopictus* from 111 geo‐referenced sites were identified and summarized in Table 2 and Additional Materials 3 and 4. The distribution of sites for the two time periods (2000–2010 and 2011–2020) across the different provinces of Cameroon are shown in Figure 1a,b and in the shiny app (https://et-ivc.shinyapps.io/Prealb/). A total of 56 sites were identified from each decade (2000–2010 and 2011–2020). Only regions in the far north recorded the absence of *Ae*. *albopictus*. The authors observed a spatiotemporal introduction of *Ae*. *albopictus* over the two decades from the southern region in the first decade to the northern region towards 2019. Areas where the vector was first identified were consistently verified by subsequent studies, indicating the establishment of the vector.

**TABLE 2:** Summary of *Ae*. *albopictus* presence and absence sites by year and publication.

Authors	Title of paper	Year collected	Num. sites	Vector presence	Vector absence	Stage(s)
Mayi et al. (2020)	Habitat and Seasonality Affect Mosquito Community Composition in the West Region of Cameroon	2019	3	3	0	Immature adult
Tedjou et al. (2020)	Patterns of Ecological Adaptation of *Aedes aegypti* and *Aedes albopictus* and Stegomyia Indices Highlight the Potential Risk of Arbovirus Transmission in Yaoundé, the Capital City of Cameroon	2018	7	7	0	Immature adult
Rodrigue Simonet et al. (2020)	Diversity and Abundance of Potential Vectors of Rift Valley Fever Virus in the North Region of Cameroon	2018	4	4	0	Adult
Nopowo et al. (2019)	Ecologie d'*Anopheles hancocki* Edwards, 1929 et étude de son implication dans la transmission du paludisme dans un village du bloc forestier sud‐camerounais	2018	1	1	0	Immature adult
Tedjou et al. (2019)	Update on the geographical distribution and prevalence of *Aedes aegypti* and *Aedes albopictus* (Diptera: Culicidae), two major arbovirus vectors in Cameroon	2017	28	21	7	Immature
Ntoumba et al. (2020)	Entomological characteristics of mosquitoes breeding sites in two areas of the town of Douala, Cameroon	2017	1	1	0	Immature
Kamgang et al. (2019)	Risk of dengue in Central Africa: Vector competence studies with *Aedes aegypti* and *Aedes albopictus* (diptera: Culicidae) populations and dengue 2 virus	2017	1	0	1	Immature
Kamgang et al. (2017)	Temporal distribution and insecticide resistance profile of two major arbovirus vectors *Aedes aegypti* and *Aedes albopictus* in Yaoundé, the capital city of Cameroon	2016	6	6	0	Immature
Mbida Mbida et al. (2017)	Nouvel aperçu sur l'écologie larvaire d'*Anopheles coluzzii* Coetzee et Wilkerson, 2013 dans l'estuaire du Wouri, Littoral‐Cameroun	2014	2	2	0	Immature
Akono Ntonga et al. (2017)	Malaria transmission and the sensitivity of aggressive mosquitoes to insecticides in a poorly urbanized area of the Deido health district in Douala (Cameroon)	2014	1	2	0	Adult
Antonio‐Nkondjio et al. (2012)	High mosquito burden and malaria transmission in a district of the city of Douala, Cameroon	2011	1	1	0	Adult
Kraemer et al. (2015)^a^	The global distribution of the arbovirus vectors *Aedes aegypti* and *Ae*. *albopictus*	2009	2	2	0	‐
		2007	26	18	8	‐
		2006	2	1	1	‐
		2004	4	0	4	‐
		2002	22	19	3	‐
Total			111	87	24	

^a^ Data by Kraemer et al. included data from 1965 to 2009. Three data points 1965(1) and 1990(2) were removed and not included in the table or analysis. The presence of *Ae*. *albopictus* in 2007 in north Cameroon was corrected to absence after an enquiry to the authors.

### Comparisons of environmental measures

After correcting for Bonferroni, there were significant differences between *Ae*. *albopictus* presence and absence sites for all the climate variables and for canopy height and forest loss (Table 3). The most dominant land cover classifications for *Ae*. *albopictus* presence sites were Savanna (*n* = 35) and Urban (*n* = 29) and for *Ae*. *albopictus* absence sites were Croplands (*n* = 15) and Savanna (*n* = 5) (data not shown).

**TABLE 3:** Descriptive statistics of the *Aedes albopictus* presence and absence and environmental variables

	Presence	Absence	Overall	Bonferroni Mann–Whitney *U*
N	87	24	111	*p*‐value
Variable	Median (95% CI)	Median (95% CI)	Median (95% CI)	Presence–Absence
Human geography				
Population	14,700 (7480–20,400)	10,400 (4590–20,000)	12,100 (7890–17,400)	1
Population density (km^2^)	528 (283–819)	365 (166–950)	450 (286–669)	1
Urban area (%)	46.6 (28.6–51.7)	39.4 (14.8–67.1)	43.3 (31–51.7)	1
Peri‐urban area (%)	0 (0–3.57)	0 (0–1.62)	0 (0–3.23)	0.3108
Rural area (%)	48.3 (37–56.7)	59 (32.9–81.5)	48.3 (39.3–60)	1
Landscape				
Elevation (m)	687 (666–731)	454 (356–818)	686 (656–723)	1
Land cover	‐	‐	‐	‐
Vegetations/forest				
EVI	0.31 (0.29–0.32)	0.25 (0.23–0.31)	0.3 (0.28–0.32)	0.2724
Canopy height (m)	3.9 (2.9–5.1)	0.55 (0.2–2.1)	3 (2.6–4)	0.0000*
Forest loss (%)	0.31 (0.15–0.55)	0.02 (0–0.2)	0.19 (0.12–0.37)	0.0007*
Climate				
Temperature (°C)	26 (25.5–26.3)	33.9 (29–35)	26.3 (26–26.5)	0.0000*
Precipitation (mm^3^)	1770 (1660–1920)	1190 (872–1530)	1660 (1610–1770)	0.0000*
Number of wet months	9 (8–9)	6 (5–7)	8 (8–9)	0.0000*

*Note*: Extracted from a 3 km buffer around the data point. Land cover excluded from summary table. *Significant (*α* = 0.05).

### Model

There was a high level of multicollinearity among the 13 original variables. Using the variance inflation factor (vif), the authors excluded five variables in the first step. Then five more variables were removed when the authors applied a stepwise process to select the best model based on the AIC. The best‐fit model included number of wet months, the percent of peri‐urban land and altitude as explanatory variables (Table 4). The higher risk factor for the presence of *Ae*. *albopictus* is the number of wet months: an increase in 1 wet month increased the odds of *Ae*. *albopictus* presence by seven times. The increase of peri‐urban land within the 3 km buffer of study site increased the odds *Ae*. *albopictus* presence by 1.3 times. The relationship with elevation was not statistically significant but lower altitudes showed marginally greater odds of *Ae*. *albopictus* presence.

**TABLE 4:** Logistic regression analysis of risk factors influencing the absence of *Ae*. *albopictus*

Predictor	Estimate	Std. error	Odds ratio	Confidence interval	*p*‐value
Intercept	−11.89	2.93	NA	‐	0.0000
Wet months	1.94	0.48	6.97	3.26–22.8	0.0000
Peri‐urban	0.27	0.13	1.31	1.06–1.79	0.0385
Elevation	−0.002	0.001	0.99	0.995–1.00	0.0663

Plots of regression model		
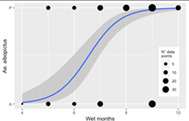	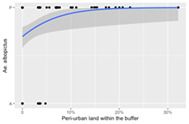	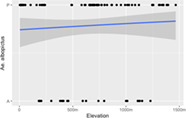

*Note*: On the bottom, A = absence, P = presence. Shade area represents the standard error.

## DISCUSSION

*Aedes*‐borne arboviruses pose a significant challenge to public health in Cameroon (Eltom et al., 2021; Simo Tchetgna et al., 2021), but little is known about the transmission systems that perpetuate these viruses in this geographical context. To begin to understand these factors, this study developed a specifically geo‐referenced database of *Ae*. *albopictus* and produced maps to highlight the geographical distributions. The examination of human/demographical and environmental variables highlighted that the amount of rainfall and the level of urbanization were important in defining the presence of *Ae*. *albopictus* in Cameroon. This is one of the few attempts to quantify the risk factors of *Ae*. *albopictus* presence in SSA, as most of the current work are global predictions or descriptive analysis of the collection sites.

We found the number of wet months and percentage of peri‐urban area around the sampling site to be most associated with *Ae*. *albopictus* presence in Cameroon. Similar variables have previously been found to be strong predictors of *Ae*. *albopictus* presence (Dickens et al., 2018; Juliano et al., 2002). More wet months provide more opportunities for breeding sites as this vector thrives in rain‐filled containers, in particular used tyres and naturally formed cavities, such as tree holes (Bonizzoni et al., 2013; Egid et al., 2021; Paupy et al., 2009; Ryan et al., 2018). The northern part of Cameroon is drier than the rest of the country potentially making it more difficult for *Ae*. *albopictus* to maintain populations. By contrast, *Ae*. *aegypti* has been reported in all the sites where *Ae*. *albopictus* was absent, indicating that *Ae*. *aegypti* might be more tolerant to drier environments (Juliano et al., 2002; Kamgang et al., 2010) or easily maintained by human water storage activities (Egid et al., 2021; Kraemer et al., 2015).

The present study's findings on the relationship between percentage of peri‐urban areas and presence of *Ae*. *albopictus* agree with other studies (Kamgang et al., 2017; Mayi et al., 2020; Paupy et al., 2010). An increase of 1% of peri‐urban area increases by 1.3× the likelihood of the vector presence. *Aedes albopictus* is an ecological flexible and opportunistic vector (Bonizzoni et al., 2013). Consequently, peri‐urban areas are ideal places for them to thrive as they originated at the fringe of forests but adapted to breed in artificial containers, and they normally feed on animals and humans, making the interface between urban and rural the perfect ecological habitat (Bonizzoni et al., 2013; Egid et al., 2021; Kamgang et al., 2012; Mayi et al., 2020; Paupy et al., 2009).

A limitation of this study is the variation in the study design of the data collated. Collection of vector data (immature stages or adults) was done using different methods, for instance, for adult mosquitoes the following methods were used: human landing catches (Akono Ntonga et al., 2017; Antonio‐Nkondjio et al., 2012; Nopowo et al., 2019), CDC light traps (Antonio‐Nkondjio et al., 2012), EVS lights (Rodrigue Simonet et al., 2020) and sweep nets (Mayi et al., 2020). Locations were not sampled over time and precision of the data points is uncertain (Akono Ntonga et al., 2017; Kamgang et al., 2017; Nopowo et al., 2019; Ntoumba et al., 2020; Rodrigue Simonet et al., 2020; Tedjou et al., 2020). The authors were able to conduct this analysis due to the advancement and availability of satellite data. However, the urbanization product, which classifies land into urban, peri‐urban and rural, was available only for 2 years, 2000 and 2015. This could be important to understand the process of urbanization in the northern part of the country, where *Ae*. *albopictus* is expanding. In addition, authors (Kamgang et al., 2017; Tedjou et al., 2019, 2020) use peri‐urban or suburbs to characterize the area of vector survey, but most did not adequately define the meaning or main characteristics of peri‐urban areas, which may differ between sites and co‐author definitions.

In contrast to other studies (Brady et al., 2014; Kraemer et al., 2015), the authors did not find temperature to be a significant predictor. This is likely due to the restricted geographic range of this analysis compared with previous studies at the global level (Brady et al., 2014; Kamal et al., 2018; Kraemer et al., 2015; Ryan et al., 2018). *Aedes albopictus* is well known to have a high tolerance for colder and mild temperatures (Juliano et al., 2002; Marini et al., 2020; Paupy et al., 2009; Thomas et al., 2012). This only becomes apparent when including sites in more extreme latitudes in the analysis, which allows *Ae*. *albopictus* to extend its range into Europe and North America (Hopperstad et al., 2021; Takumi et al., 2009). For instance, in contrast to other analysis delimitation (Tedjou et al., 2019), Rodrigue Simonet et al. (2020) found for first time *Ae*. *albopictus* in latitudes above 6° N. Further studies and molecular analysis are needed to elucidate whether *Ae*. *albopictus* is established in this latitude for first time, or secondary vectors as *Ae*. *unilineatus* are the ones circulating in this part of the country. This vector expansion to country north should be explored in future studies. The authors also have not found a relationship between changes in the land use, forest loss or the height of canopy. This might be due to the juxtaposition of different spatiotemporal resolutions and the absence of data points in the same place over time. As opposed to climatic factors that change little in small geographical area, land use can change greatly, especially around urban areas in a context of rapid urbanization (United Nations, Department of Economic and Social Affairs, 2019). However, as seen in this study, even in the same country, data collection is not performed in the same location over time. Thus, evaluation of the impact of land use and land cover at fine scale at the same location over time is needed to understand the impact on vector distribution and helps to facilitate control strategies.

## CONCLUSION

This is one of the first studies to quantify the influence of human and physical geography on *Ae*. *albopictus* in SSA. The length of wet months and the extent of peri‐urban areas were found to be important risk factors in Cameroon, which provides some insights into the local vector ecology. However, for the national arbovirus disease control programmes to move forward, more standardized methodologies over time and space are needed to be able to better quantify the risk factors. This may facilitate appropriately targeted public health messages and vector control strategies.

## AUTHOR CONTRIBUTIONS

Tiago Canelas, Edward Thomsen, Basile Kamgang and Louise A. Kelly‐Hope conceived and designed the analysis; Tiago Canelas collected the data; Tiago Canelas performed the analysis; Tiago Canelas, Edward Thomsen, Basile Kamgang and Louise A. Kelly‐Hope wrote the paper.

## FUNDING INFORMATION

Tiago Canelas and Edward Thomsen are funded by the Medical Research Council of the UK (grant number MR/P027873/1) through the Global Challenges Research Fund.

## CONFLICT OF INTEREST

The authors declare that the research was conducted in the absence of any commercial or financial relationships that could be construed as a potential conflict of interest.

## Supporting information


**Appendix S1:** Supporting Information.

**Appendix S2:** Supporting Information.

**Appendix S3:** Supporting Information.

**Appendix S4:** Supporting Information.

## Data Availability

All datasets used for this work are publicly available, in the manuscript or in the additional material.
