# Supplementary material for: Demographic and environmental factors associated with the distribution of Aedes albopictus in Cameroon
Source: Med Vet Entomol. 2022 Oct 20;37(1):143–51. doi: 10.1111/mve.12619 (PMC10092813; doi:10.1111/mve.12619)
Supplement: Supplementary file 1 — Appendix S1: Supporting Information. [file MVE-37-143-s003.docx]

# Additional material 1

Literature search strategy:

**Databases:** PubMed and Web of Science

**Search date**: May 2021

**Keywords:** (Cameroon) AND (Aedes); (Cameroon) AND (Aedes aeypti); (Cameroon) AND (Aedes albopictus); (Cameroon) AND (arbovir*); (Cameroon) AND (dengue); (Cameroon) AND (chikungunya).

**Peer Reviewed Published Date:** All time range available in the databases

**Language:** Any
