# Supplementary material for: Demographic and environmental factors associated with the distribution of Aedes albopictus in Cameroon
Source: Med Vet Entomol. 2022 Oct 20;37(1):143–51. doi: 10.1111/mve.12619 (PMC10092813; doi:10.1111/mve.12619)
Supplement: Supplementary file 2 — Appendix S2: Supporting Information. [file MVE-37-143-s004.docx]

# Additional material 2

Variable’s description with full sources.

| **VARIABLE** | **DESCRIPTION** | **SOURCE** |
| --- | --- | --- |
| Population | People per pixel adjusted UN estimated | WorldPop (Worldpop, 2021a) |
| Population density | Number of people per pixel divided by pixel size | WorldPop (Worldpop, 2021b) |
| Degree of urbanisation | Classification on urban, peri-urban or rural area within the pixel. | GHSL (Pesaresi *et al.*, 2019) |
| Elevation | Elevation above sea level | SRTM (Earth Resources Observation and Science Center, 2000) |
| Land cover | Land cover types. We used the Annual International Geosphere-Biosphere Programme (IGBP) classification | MODIS (Sulla-Menashe and Friedl, 2021) |
| Enhanced Vegetation Index | Yearly averaged EVI by pixel | MODIS (Schaaf and Wang, 2015) |
| Forest canopy height | Footprint-based measurements of vegetation structure, including forest canopy height in meters | UMD GLAD (Potapov *et al.*, 2021) |
| Forest loss | Changes of trees (>5m) in a pixel over a year | UMD GLAD (Hansen *et al.*, 2013) |
| Land Surface Temperature | Monthly and yearly averaged temperature by pixel. | MODIS (Wan, Hook and Hulley, 2015) |
| Rainfall | Monthly and yearly total rainfall by pixel. | CHIRPS (Funk et al., 2015) |
| Wet months | Number of months with more rainfall than 60mm^3^. Köppen classification (Peel, Finlayson and McMahon, 2007) | CHIRPS (Funk *et al.*, 2015) |

**References:**

Earth Resources Observation and Science Center (2000) *Shuttle Radar Topography Mission (SRTM) 1 Arc-Second Global*. Available at: <https://www.usgs.gov/centers/eros/science/usgs-eros-archive-digital-elevation-shuttle-radar-topography-mission-srtm-1-arc?qt-science_center_objects=0#qt-science_center_objects> (Accessed: 5 Jan 2022).

Funk, C. *et al.* (2015) ‘The climate hazards infrared precipitation with stations - A new environmental record for monitoring extremes’, *Scientific Data*, 2, pp. 1–21. doi: 10.1038/sdata.2015.66.

Hansen, M. C. *et al.* (2013) ‘High-Resolution Global Maps of 21st-Century Forest Cover Change’, *Science*, 342(6160), pp. 850–853. doi: 10.1126/science.1244693.

Peel, M. C., Finlayson, B. L. and McMahon, T. A. (2007) ‘Updated world map of the Köppen-Geiger climate classification’, *Hydrology and Earth System Sciences*, 11(5), pp. 1633–1644. doi: 10.5194/hess-11-1633-2007.

Potapov, P. *et al.* (2021) ‘Mapping global forest canopy height through integration of GEDI and Landsat data’, *Remote Sensing of Environment*, 253, p. 112165. doi: 10.1016/j.rse.2020.112165.

Schaaf, C. and Wang, Z. (2015) *MCD43A4 MODIS/Terra+Aqua BRDF/Albedo Nadir BRDF Adjusted Ref Daily L3 Global - 500m V006 [Data set].*, *NASA EOSDIS Land Processes DAAC*. doi: 10.5067/MODIS/MCD43A4.006.

Sulla-Menashe, D. and Friedl, M. (2021) *MCD12Q1 MODIS/Terra+Aqua Land Cover Type Yearly L3 Global 500m SIN Grid V006 [Data set]*, *NASA EOSDIS Land Processes DAAC.* doi: 10.5067/MODIS/MCD12Q1.006.

Wan, Z., Hook, S. and Hulley, G. (2015) *MOD11A2 MODIS/Terra Land Surface Temperature/Emissivity 8-Day L3 Global 1km SIN Grid V006 [Data set]*, *NASA EOSDIS Land Processes DAAC*. Available at: <https://lpdaac.usgs.gov/products/mod11a2v006/#citation> (Accessed: 5 Jan 2022).

Worldpop (2021a) *Population counts*. Available at: <https://www.worldpop.org/project/categories?id=3> (Accessed: 5 Jan 2022).

Worldpop (2021b) *Population density*. Available at: <https://www.worldpop.org/project/categories?id=18> (Accessed: 5 Jan 2022).
