# Supplementary material for: Demographic and environmental factors associated with the distribution of Aedes albopictus in Cameroon
Source: Med Vet Entomol. 2022 Oct 20;37(1):143–51. doi: 10.1111/mve.12619 (PMC10092813; doi:10.1111/mve.12619)
Supplement: Supplementary file 4 — Appendix S4: Supporting Information. [file MVE-37-143-s001.docx]

# Additional material 4

Distribution of sites and presence by province for the period 2000 - 2010 and 2011 - 2020.

|  | 2000 - 2010 | | | 2011 - 2020 | | |
| --- | --- | --- | --- | --- | --- | --- |
| ***Ae. albopictus*** | **Presence** | **Absence** | **Total** | **Presence** | **Absence** | **Total** |
| Adamaoua | 2 | 4 | 6 | 2 | 5 | 7 |
| Centre | 8 | 1 | 9 | 17 | 0 | 17 |
| Est | 7 | 0 | 7 | 3 | 0 | 3 |
| Extreme – Nord | 0 | 5 | 5 | 0 | 1 | 1 |
| Littoral | 8 | 0 | 8 | 8 | 0 | 8 |
| Nord | 0 | 6 | 6 | 4 | 2 | 6 |
| Nord – Ouest | 2 | 0 | 2 | 1 | 0 | 1 |
| Ouest | 6 | 0 | 6 | 6 | 0 | 6 |
| Sud | 2 | 0 | 2 | 3 | 0 | 3 |
| South - Ouest | 5 | 0 | 5 | 3 | 0 | 3 |
| **Total** | **40** | **16** | **56** | **47** | **8** | **55** |
| Douala | 4 | 0 | 4 | 6 | 0 | 6 |
| Yaoundé | 4 | 1 | 5 | 12 | 0 | 12 |
| Garoua | 2 | 3 | 5 | 2 | 1 | 3 |
| **Total** | 9 | 4 | 13 | 20 | 1 | 21 |
